# Male production in green sea turtles under a feminizing climate: A validated model of sex ratios with temperature‐dependent sex determination

**DOI:** 10.1111/1365-2656.70334

**Published:** 2026-08-11

**Authors:** Marc Girondot, Emily Turla, Kirsty Scott, Jeanette Wyneken

**Affiliations:** ^1^ Ecologie, Société, Evolution Université Paris‐Saclay, CNRS, AgroParisTech Gif‐sur‐Yvette France; ^2^ Department of Biological Sciences Florida Atlantic University Boca Raton Florida USA; ^3^ Marine Science Program, Division of Biological and Environmental Science and Engineering King Abdullah University of Science and Technology Thuwal Kingdom of Saudi Arabia

**Keywords:** climate, feminization, marine turtles, masculinization, temperature, temperature‐dependent sex determination

## Abstract

1. Green sea turtles (*Chelonia mydas*) are globally distributed and use tropical and subtropical beaches for nesting. Green turtles exhibit temperature‐dependent sex determination (TSD), a mechanism by which incubation temperature during a critical developmental window determines offspring sex. As global temperatures rise, many nesting populations could be producing increasingly female‐biased sex ratios, raising concerns about long‐term population viability. Our ability to assess demographic risks has been hindered by the absence of validated models that accurately convert field‐recorded nest temperatures into sex ratios. In this study, we combine high‐resolution temperature data from 62 monitored nests with known hatchling sex ratios (*n* = 594 individuals) to develop and validate a predictive model for sex ratio in green turtles.
2. We developed several methodological innovations to study the impact of temperature‐dependent sex determination (TSD) under natural conditions. First, we developed a novel approach to quantify spatial temperature heterogeneity (*H*, range between the 2.5th and 97.5th percentiles of the pairwise differences of nest temperatures recorded at the same time) on a nesting beach. The median heterogeneity of the beach is 1.58°C (95% credible interval: 1.52–1.64°C). Second, using a Bayesian mixed‐model framework and experimental data from 1480 embryos incubated at constant temperatures under different moisture conditions, we detected genetic and/or maternal differences among clutches in the thermal sensitivity of sex ratio estimates, but no effect of Regional Management Units (RMU). Third, we fitted nine models to estimate field sex ratios; the best‐performing model combined a thermal reaction norm of sexualization with a sensitivity function across the thermosensitive period (TSP), showing that both the timing and magnitude of temperature exposure influence sex determination.
3. Our results indicate that males can be produced even under apparent female‐producing conditions, due to temperature heterogeneity, temporal autocorrelation of nest temperatures that increased sex ratio variance and non‐linear sensitivity to male‐producing temperatures. This study establishes a new standard for predicting sex ratios in reptiles with TSD, offering critical insights for population assessments and adaptive conservation planning under changing climatic conditions.

## Graphical abstract

This study introduces validated methods to predict sex ratios in wild Green sea turtle (*Chelonia mydas*) nests. It quantifies beach‐scale temperature heterogeneity, incorporates clutch‐level variation in thermal sensitivity and models temperature effects across the thermosensitive period, demonstrating how temporal temperature dynamics and spatial variability allow male production even under predominantly female‐producing conditions.

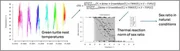

## 1 INTRODUCTION

Green sea turtles (*Chelonia mydas*) exhibit temperature‐dependent sex determination (TSD), a mechanism in which the ambient temperature during egg incubation determines the sex of the offspring (Mrosovsky & Pieau, 1991; Standora & Spotila, 1985). This trait makes them vulnerable to climatic shifts, as rising global temperatures could lead to increasingly female‐biased sex ratios in nesting populations (Jensen et al., 2018), but could compensate for the lower hatching success due to high temperatures (Santidrián Tomillo & Spotila, 2020). Such demographic shifts raise concerns for the long‐term viability of populations since an extreme female bias could limit reproductive output and reduce genetic diversity of populations over time. Monitoring and understanding current sex ratio patterns are therefore essential not only for predicting future population dynamics but also for informing targeted conservation strategies, such as nest shading or relocation. Despite growing attention on the effects of climatic shifts on sea turtle reproduction, empirical data on sex ratios remain sparse.

The green sea turtle (hereafter green turtle) has the highest number of known nesting beaches of all sea turtle species (Seminoff, 2023), using beaches up to 35° North and South of the equator. Given the green turtle's broad nesting range and the influence of natal philopatry, it remains unclear how this species continues to produce males and females at frequencies compatible with the long‐term persistence of the population. Recent studies on the resilience of marine turtles to climate warming in spite of having TSD have focused on phenological shifts (Fuentes et al., 2024; Witt et al., 2025) and colonization of new nesting habitats (Carreras et al., 2018; Santidrián Tomillo et al., 2023). For phenological shifts, the conclusions can be contradictory depending on species and populations. For example, loggerhead sea turtles (*Caretta caretta*) in Northern Cyprus are currently compensating for the effects of rising temperatures on offspring sex ratios through a change in the phenology of nesting (Witt et al., 2025). However, the rate of temperature change is too fast for six out of seven other loggerhead sea turtle populations, suggesting they cannot keep pace with the current rate of warming (Kirkham et al., 2025; Monsinjon et al., 2019). For spatial shifts, two different phenomena can be used to maintain viable sex ratios. Sporadic nesting events close to developmental foraging grounds may be a mechanism used to overcome philopatry constraints, increase the dispersal capabilities of the species and adaptively respond to changing environments (Carreras et al., 2018). Another approach may be the increased use of minor nesting beaches, which could function as climate refuges, because temperatures may favour embryo success and a male sex ratio (Santidrián Tomillo et al., 2023; Stokes et al., 2024).

Prior to hypothesizing complex phenological or spatial shifts, investigating how the nesting beach influences sex ratios in marine turtles is an essential first step. The relationship between field temperatures and resulting sex ratios remains limited (Fuentes et al., 2017), and intra‐beach variability or its impact on sex ratio is seldom assessed (Sugimoto et al., 2025). To do this, only a rough method to convert time series of temperature into sex ratio has been used without any validation using observed sex ratios. The only validated model available to convert a time series of temperatures into sex ratio is a semi‐mechanistic model that estimates a constant‐temperature equivalent (CTE) within the thermosensitive period of development for sex determination (TSP). The sex ratio can then be estimated by applying this CTE to a model of thermal population reaction norm of sex ratio. This model has only been parameterized for the loggerhead sea turtle, *C. caretta*, so far (Monsinjon et al., 2022).

Our understanding of the impact of climatic changes on green turtles has been limited by the absence of large temporal and spatial scale datasets of true sex ratio. This data gap is due to ethical constraints because the only way to determine sex was by euthanizing hatchlings to examine their gonads. As a consequence, a validated model to estimate sex ratio was also lacking. This limitation stems largely from a lack of datasets that combine high‐resolution temperature records within nests and known sex ratios from monitored clutches. To address this gap, our study aims to assess the primary sex ratio of green turtles, providing insights into population demographics under environmental change. Furthermore, our findings may support the broader application of a semi‐mechanistic model of sex ratio to other turtle or tortoise species with TSD (Krueger et al., 2025).

## 2 MATERIALS AND METHODS

### 2.1 Field data and observed sex ratio

Clutch temperatures were recorded of the total incubation for 67 green turtle nests at two nesting beaches in Boca Raton (Latitude 26.350° N, Longitude 80.069° W) and Jupiter (Latitude 26.931° N, Longitude 80.069° W), in Florida, USA, between 2019 and 2024. The sands on these beaches are moderately sorted particles of quartz and shell. Temperatures were recorded during incubation throughout the nesting season; early, middle and late parts of the season were selected based on historical nesting and hatchout data (Lolavar & Wyneken, 2015). Nests that were at risk of washout were not instrumented for this study. HOBO U22‐001 temperature dataloggers (accuracy ±0.2°C, resolution 0.02°C; Onset Computer Corp., Bourne, MA USA) were placed in the middle of the clutch (~55 cm deep) and set to record temperatures at a frequency of 15 min except for one nest in 2021 (30 min) and one in 2024 (60 min). We sampled 1 to 10 turtles from 64 of these 67 nests (mean = 9.3; SD = 1.9; total sexed juveniles = 594); which were raised for several months in seawater tanks until they reached a sufficient size for safe laparoscopy (minimum size 120 g for *C. mydas*) after Wyneken et al. (2007). The choice of sexing 10 turtles per nest represents a compromise between accurately representing the sex ratio within each nest, the need to obtain mixed‐sex nests in the sample to increase the power for modelling sex ratio and the cost of rearing and sexing 594 juveniles. The observed probability of a mixed‐sex nest is 12/61 = 0.2. The probability that all nests in a sample of *n* nests are unisexual is therefore ${\left(1-0.2\right)}^{n}$. Let's take three examples, with n=6, 15, and 61 nests. If n=6, the probability that all nests would be unisexual would be 0.26. If n=15, the probability that all nests would be unisexual would be still 0.04. In contrast, when 10 turtles are sexed per nest for 61 nests, the probability that all nests are unisexual drops to 0.000001.

Post‐hatchlings were released offshore no less than 7 days after sex identification, as boat transport became available. Twelve dead post‐hatchlings from the sample laboratory colonies were sexed following necropsy and were included in the sex ratio modelling. Gonad biopsies were taken from a subset of the laparoscopically sexed turtles to ensure sex verification was consistent and validated. Data from three of the 67 nests (*n* = 22 sexed post‐hatchlings) were not included in embryo growth or sex ratio modelling because the nests were predated or temperature recording stops during incubation. Thus, the recorded temperatures of these nests were not reliable. In summary, we have used temperature data from 64 nests to model embryo growth and from 61 nests (572 sexed post‐hatchlings) to model sex ratio (Supporting Information S1: Information of monitored nests).

We estimated nest temperature heterogeneity (*H*) at the scale of the beach by comparing overlapping temperature time series recorded in different nests. To avoid the influence of metabolic heating, we used only the first half of each time series. Whenever temperatures were recorded simultaneously in x nests, we calculated the pairwise temperature differences between all nests (i.e. $x\left(x-1\right)/2$ comparisons). Temperature heterogeneity (*H*) was defined as the range between the 2.5th and 97.5th percentiles of these differences, representing the interval containing 95% of the observations. We used percentiles rather than the full range to reduce the influence of outliers. Because smaller samples tend to underestimate variation, we corrected for this bias by modelling the estimated 2.5th and 97.5th percentiles for each time bin as a function of the number of overlapping time series.

This relationship was modelled with a scaled logistic function, where the upper asymptote represents the expected heterogeneity as the number of time series approaches infinity. The model was fitted using a Bayesian approach with wide uniform priors and a Gaussian likelihood, running 50,000 iterations with thinning every 10 steps after a warm‐up of 50,000 iterations to reduce autocorrelation. The standard deviation (*SD*) of the Gaussian distribution was modelled as a linear function of heterogeneity (*H*), allowing for heteroscedasticity: SD=a×H+b, where *a* and *b* are fitted coefficients. This method is scripted in the function *HeterogeneityNests*() available in R package *embryogrowth* available in CRAN (Girondot, 2025).

Panel autocorrelation of all time series of temperatures recorded with a 15‐min time lag was estimated using psacf() from collapse R package (Krantz, 2024). The psacf() function standardizes (scales and centres) the panel‐series within each nest and then computes the lagged correlations (covariance divided by lag 0 variance).

Air temperature at 2 m altitude was extracted from the European Centre for Medium‐Range Weather Forecasts ERA‐5 database at the nearest location (longitude −80°, latitude 26.5°; WGS 84). ERA5 is the 5th generation ECMWF atmospheric reanalysis of the global climate covering the period from January 1950 to present (Copernicus Climate Change Service (C3S), 2017). ERA5 provides hourly estimates of a large number of atmospheric, land and oceanic climate variables using 4D‐Var data assimilation and model forecasts.

### 2.2 TSD thermal reaction norm

A TSD thermal reaction norm refers to representation of the relationship between constant incubation temperatures and sex ratio (Krueger et al., 2025). It is sometimes referred to as the thermal reaction norm of sex ratio (Abreu‐Grobois et al., 2020). The concept of thermal reaction norms permits studying the effect of temperatures on various biological systems (Dobzhansky, 1955). A reaction norm describes the range of phenotypes a single genotype (G) can express across an environmental gradient (E). Here, the combined pattern across multiple genotypes was studied and it is sometimes named the population reaction norm or mean reaction norm across genotypes. Strictly speaking, the population reaction norm is a representation of the G × E interaction (Via & Lande, 1985). For simplicity, here we used ‘reaction norm’ as an equivalence of the population reaction norm.

#### 2.2.1 Data at constant temperatures

We used the Reptilian Offspring Sex and Incubation Environment (ROSIE, v. 1.03) database (Krueger & Janzen, 2022). Each 1135 *C. mydas* dataline was validated by checking the original publication and duplicated data were removed. Only sexed embryos or juveniles verified using histology of the gonad from constant temperature incubation experiments (less than 0.5°C of fluctuation) were retained. Data with unstable incubation temperatures (e.g. Mrosovsky et al., 1984) were also removed from our analysis as well as all eggs manipulated by chemical addition in ecotoxicological studies. Individuals with ovotestes were counted as males and ambiguous data were discarded from the analysis (see Supporting Information S2 for a complete list of discarded data).

A total of 1480 eggs from six *Chelonia mydas* regional management units (RMUs) (NA—North Atlantic; SA—South Atlantic; NWI—Northwest Indian; EISA—East Indian and Southeast Asia; NCP—North Central Pacific; SWP—Southwest Pacific in Wallace et al., 2023) incubated at 107 constant incubation temperatures and sexed in 12 studies have been retained here (see Supporting Information S3 for the complete list). When the clutch identity was not available in the original publication, it was replaced by the publication reference to retain relevance to RMU‐level information.

#### 2.2.2 Statistical description of TSD pattern

The patterns of TSD were fitted using a sigmoid model for *sr* being the sex ratio upon the *T* temperature; *sr*(*T*) (Equation 1; the definition of parameters is the same in all the equations). We implemented the asymmetrical model named flexit** from the *embryogrowth* package (Girondot, 2025), which is an enhanced version of the *flexit* model (Abreu‐Grobois et al., 2020). The original *flexit* model used two parameters (*K*_1_ and *K*_2_) to model asymmetry around the pivotal temperature (*P* parameter), and S parameter was the slope of sex ratio change at P. We changed the parametrization to three parameters: *P*, the pivotal temperature, SL the distance in°C from P to a *l* = 0.05 male relative frequency and SH the distance in°C from P to a 1−l = 0.95 male relative frequency. The advantage of this parametrization is that all three parameters have the same unit (°C) and can be directly compared. The transitional range of temperatures (TRT) is the range of constant incubation temperatures producing both sexes between limits *l* and 1−l. The posterior predictive TRT is then SL+SH.

(1) $$\mathrm{sr}\left(T\right)={\left(1+e^{\frac{\log\;\left(\frac{l}{\left(1-l\right)}\right)\;\left(P-T\right)}{\mathrm{SL}\times \mathrm{QBT}+\mathrm{SH}\times \left(1-\mathrm{QBT}\right)}}\right)}^{-1}\;\text{with}\;\mathrm{QBT}=1\;\text{if}\;T<P\;\text{or}\;\mathrm{QBT}=0\;\text{if}\;T\geq P.$$

The three parameters of the flexit** model (*P*, *SL*, *SH*) have been fitted using a Bayesian MCMC model with a binomial likelihood (Abreu‐Grobois et al., 2020). This model was implemented in the *embryogrowth* R package (version >10.1) and has been scripted in STAN (Stan Development Team, 2023) to include random factors. Nested random factors were RMU effect (Wallace et al., 2023) and Clutch effect (Krueger & Janzen, 2022). The effect of random factors was applied in the three parameters (*SL*, *SH*, and *P*) or only to *P*. The tested random effects are: 1 (no random effect), 1 + (1 | RMU), 1 + (1 | RMU/Clutch), and 1 + (1 | Clutch). Priors for P were chosen from a normal distribution $N\left(30,1\right)$ based on the known distribution of pivotal temperatures in turtles (Hulin et al., 2009) and a lognormal distribution (meanlog = −0.2, sdlog = 0.8) for SL and SH. The priors for the standard deviations of the random effects have been chosen from a Student's t‐distribution with nu = 3, mu = 0 and sigma = 4.4 (default proposed by STAN software). Models were run for 130,000 iterations with 30,000 iterations for warm‐up and were thinned by 10 iterations to remove autocorrelation.

Model selection was performed using the Expected Log Predictive Density (ELPD) estimated through leave‐one‐out cross‐validation (LOO) (see Supporting Information S4: Common statistical procedures and list of parameters). The ELPD provides an information‐theoretic criterion that balances goodness‐of‐fit with model complexity, effectively penalizing models that overfit the data (Vehtari et al., 2017, 2024).

### 2.3 Model of sex ratio from field data

#### 2.3.1 Thermal reaction norm of embryo growth

We modelled the progression of the size (straight carapace length, SCL) of green turtle embryos throughout incubation using an embryonic growth model that combines the thermal reaction norm for growth rate and a growth function (Fuentes et al., 2017).

Biological temperature‐dependent reaction norms based on Arrhenius' and Eyring's equations were formulated by Schoolfield et al. (1981). Equation 2 shows the 4‐parameter version; the 6‐parameter version can be found in the original publication. Both equations are tested here.

(2) $$r\left(T\right)=\frac{\rho_{\left(298\;K\right)}\frac{T}{298}\exp\left[\frac{{∆H}_{A}^{\neq }}{R}\left(\frac{1}{298}-\frac{1}{T}\right)\right]}{1+\exp\left[\frac{∆H_{H}}{R}\left(\frac{1}{{T_{1/2}}_{H}}-\frac{1}{T}\right)\right]}$$

T being the temperature in *K* (298 *K* = 24.85°C), $r\left(T\right)$ is the development rate at temperature T(time^−1^), *R* the universal gas constant (J K^−1^ mol^−1^), ${∆H}_{A}^{\neq }$ the enthalpy of activation of the reaction catalysed by the enzymes (J mol^−1^), $∆H_{H}$ the change in enthalpy associated with the high‐temperature inactivation of the enzymes (J mol^−1^) and $T_{1/2_{H}}$ is the temperature in K at which the enzymes are 1/2 active and 1/2 high‐temperature inactive.

The growth of embryos was modelled using a modification of the Gompertz model proposed by Laird (1964). Let $X\left(0\right)$ be the embryo size or mass at the time of oviposition (time = 0), *r*(*T*) is the growth rate and *K* is the asymptotic size with $X\left(t\right)=K$.

(3) $$X\left(t\right)=K\;\exp\left(\ln\left(\frac{X\left(0\right)}{K}\right)\exp\left(-r\left(T\right)\;t\right)\right)$$

Note that hatching generally occurs before the time when the embryo reaches a size or mass *K*. The *K* parameter here can be viewed simply as a way to account for slower growth at the end of incubation.

Parameters $X\left(0\right)$ = 0.34 mm (SE = 0.19 mm) and *K* = 47 mm (SE = 3.59 mm) and then *rK*=*K*/(SCL at emergence) = 1.209 were determined based on the growth model applied on the turtles' (freshwater and marine) developmental table (Girondot et al., 2018). Parameters $T_{1/2_{H}}$, $∆H_{H}$, ${\mathrm{\Delta H}}_{A}^{\neq }$ and $\rho_{\left(298\;K\right)}$ were fitted using field incubation temperatures (*n* = 64 nests; two nests have been removed from total dataset because dataloggers were displaced by predators). The SCL (mean = 49.73 mm, SD = 2.74) of 643 hatchlings was measured from the 67 monitored nests that incubated at the nesting beaches in Boca Raton and Jupiter, Florida. The model with six parameters was fitted using the same conditions. The 4‐ and 6‐parameter models were fitted using Bayesian statistics with wide uniform priors and compared using ELPD (see Supporting Information S4: Common statistical procedures and list of parameters).

#### 2.3.2 Model for sex ratio estimation

The thermosensitive period of development for sex determination (TSP) is defined as the interval of development when the variation of temperature has some significant effect on sex ratio (Mrosovsky & Pieau, 1991). The TSP begins with the formation of the genital ridge, which is the onset of the gonad formation (stage 23) and ends at stage 27 using the embryonic stages based on the developmental table from Miller (1985). The exact delimitation of embryological stages is subject to uncertainty because it was based on incubation of few eggs (Yntema & Mrosovsky, 1982). To take into account this uncertainty, the TSP limits are set as fitted parameters: BeginTSP and EndTSP parameters with BeginTSP < EndTSP (each defined from 0 at egg laying to 1 at hatching in logit scale). The SCL at beginning and end of TSP are $\text{BeginTSP}\times {\mathrm{SCL}}_{\text{Hatching}}$ and $\text{EndTSP}\times {\mathrm{SCL}}_{\text{Hatching}}$, respectively.

The thermal reaction norm for sexualization (*TRNS*) links incubation temperatures during the TSP and the molecular mechanisms that determine sexualization of the developing embryo. The *TRNS* is then the differential capacity of a temperature to sexualize an embryo. We model the *TRNS* using the general equation describing thermal reaction norms (Schoolfield et al., 1981) (Equation 2). The parameters of the *TRNS* are then ${\text{TRNS}.∆H}_{A}^{\neq }$, $\text{TRNS}.∆H_{H}$ and $\text{TRNS}.T_{1/2_{H}}$. $\text{TRNS}.\rho_{\left(298\;K\right)}$ is fixed to 100 as only relative values are of interest (see Equation 2 for TRNS and Equation 4 for its use in CTE estimate).

The sensitivity of sexualization during the thermosensitive period of development for sex determination (S‐TSP) is the differential effect of temperatures during the TSP. It is modelled as the density of a beta distribution $Β\left(t;\text{shape}1,\text{shape}2\right)$ with 0 < *t* < 1, shape1 >0 and shape2 >0. The time *t* = 0 is the beginning of TSP and *t* = 1 is the end of TSP defined by BeginTSP and EndTSP parameters (see previous paragraph). Although the use of a beta distribution has no mathematical justification, it has empirical utility due to its large shape flexibility to model the influence of temperature during the TSP, where different shapes like U, n, −, J or L can be modelled depending on the values of the two parameters shape1 and shape2.

The parameters of the sexualization models are obtained by fitting observed sex ratios with estimated sex ratios obtained by conversion of the recorded temperatures in natural nests into CTE using the weighting scheme:

(4) $$\mathrm{CTE}=\frac{\sum_{t=\text{BeginTSP}}^{\text{EndTSP}}\left(T_{t}\times ∆t\;\mathrm{ime}\times \text{GrowthRate}\;\left(T_{t}\right)\times \text{TRNS}\;\left(T_{t}\right)\times S-\mathrm{TSP}\;\left(t\right)\right)}{\sum_{t=\text{BeginTSP}}^{\text{EndTSP}}\left(∆\text{time}\times \text{GrowthRate}\;\left(T_{t}\right)\times \text{TRNS}\;\left(T_{t}\right)\times S-\mathrm{TSP}\;\left(t\right)\right)}$$

The sex ratio predictions were based on time‐weighted (∆time in Equation 4) or growth‐weighted ($\text{GrowthRate}\left(T_{t}\right)$ in Equation 4) temperatures ($T_{t}$ in Equation 4).

The expected sex ratio is then *sr*(CTE) as given by Equation 1. Note the *t* in *S‐TSP*(*t*) is the standardized time during TSP being 0 at *BeginTSP* and 1 at *EndTSP*.

In summary, *BeginTSP* and *EndTSP* control the position of the TSP to take into account uncertainty on the end of TSP (default is from stage 23 to the centre of stage 27). The *TRNS* measures the strength of different temperatures to influence sex determination (default considers no difference in sexualization from different temperatures) and *S‐TSP* measures the relative influence of temperatures during the TSP (default is considered the same influence of temperature during any part of the TSP).

The different models' results were tested using AICc (Akaike Information Criterion corrected for finite sample size) (Burnham & Anderson, 2002) and only the selected model based on AICc was fitted using a Bayesian model.

## 3 RESULTS

### 3.1 Field data and observed sex ratio

Nest temperatures were collected from the middle of the clutch and ranged from 27°C to 37°C (Figure 1a). The nest temperature profiles follow the same pattern as 10‐day average air temperature recorded at 2 m altitude in close proximity to the nesting beach; nest temperatures (coloured lines in Figure 1a) increased by +5°C above air temperatures (black line in Figure 1a). The observed sex ratio for the monitored clutches is also shown in Figure 1a (black points at the top of the temperature profiles). The sex ratio over the time series was generally female‐biased but some males were produced. A temporal pattern of male production was observed, with males predominantly produced in nests laid later in the season (Bayesian GLM with binomial distribution, logit link and flat prior; ordinal date effect = 0.03, 95% credible interval [0.02, 0.04]).

**FIGURE 1:**
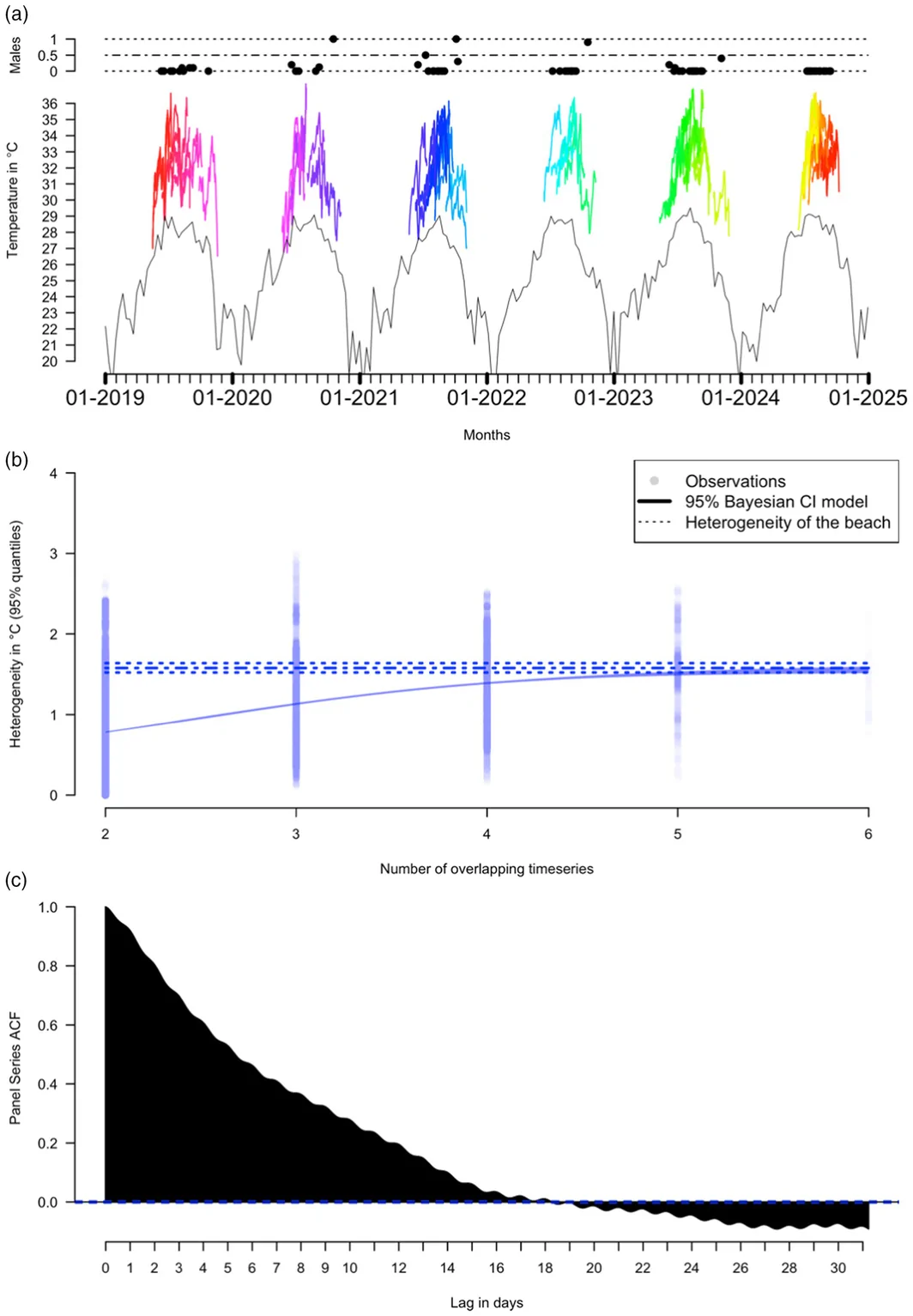
(a) Temperature records of the 64 nests monitored on Florida beaches (coloured plot lines), 10‐day average air temperature at 2 m high (plain black lines) and male frequency for 61 of these 64 nests (solid points at top, x‐position is the middle of incubation for each nest). (b) Each point represents the observed temperature difference between two nest temperatures recorded at the same time. The horizontal lines are the median (long dashes) and 95% credible interval (short dashes) of the beach temperature heterogeneity measured as nest temperature 95%‐interquantiles. The model converged on a median heterogeneity of 1.58°C (95% credible interval: 1.52–1.64°C) for the beach. (c) Temperature autocorrelation for the 62 time series with 15‐min time‐laps recording. Positive autocorrelations are observed up to 17 days.

The temperatures during each nesting season are variable. The main factor influencing the yearly pattern of temperatures is not the maximum temperatures, which are relatively constant between years, but the temporal width of each annual temperature peak (black line in Figure 1a). As an example, in 2024, the warmest air temperature averages persisted longer than in previous years.

The heterogeneity (*H*, range between the 2.5th and 97.5th percentiles of the pairwise differences of nest temperatures) of first‐half nest temperatures (to exclude metabolic heating) is shown in Figure 1b. As expected, the more series of temperatures that are included, the higher the heterogeneity among nests. The model converged to a median heterogeneity in nest temperatures of 1.58°C (95% credible interval: 1.52–1.64°C) for the beach, with a maximum heterogeneity of 2.16°C when accounting for the fitted standard deviation (*SD*). Temporal panel autocorrelation shows a positive autocorrelation up to 17 days (Figure 1c). A total of 594 post‐hatchlings were sexed from 64 nests, including the nests being predated, with 50 males and 544 females. The male relative frequency per nest was 0.08 (SD = 0.22). This large SD indicates a significant deviation from a binomial distribution with more observed extreme sex ratios than expected (expected SD based on 10,000 random replicates = 0.10; a value ≥0.22 is never observed in these 10,000 replicates).

### 3.2 TSD thermal reaction norm

The sex ratios of 1480 embryos incubated under constant temperature conditions were modelled using a Bayesian flexit** model, either without random effects or incorporating random effects structures as follows: 1 + (1 | RMU), 1 + (1 | RMU/Clutch) and 1 + (1 | Clutch). The models are ranked using the probability that each model is the best among the studied ones using difference of expected log pointwise predictive density to identify which model best predicts novel data (Figure 2a). The model with random Clutch effects on *P*, *SL* and *SH*, model C(P, S) in Figure 2a, has 96% chance to be the best model among the seven tested ones based on difference of expected log pointwise predictive density (Vehtari et al., 2017). The addition of a RMU random effect on *P*, *SL* and *SH*, model R/C(P, S) in Figure 2a, degraded the performance indicating an overparametrization: all the RMU information was already present in Clutch effect.

**FIGURE 2:**
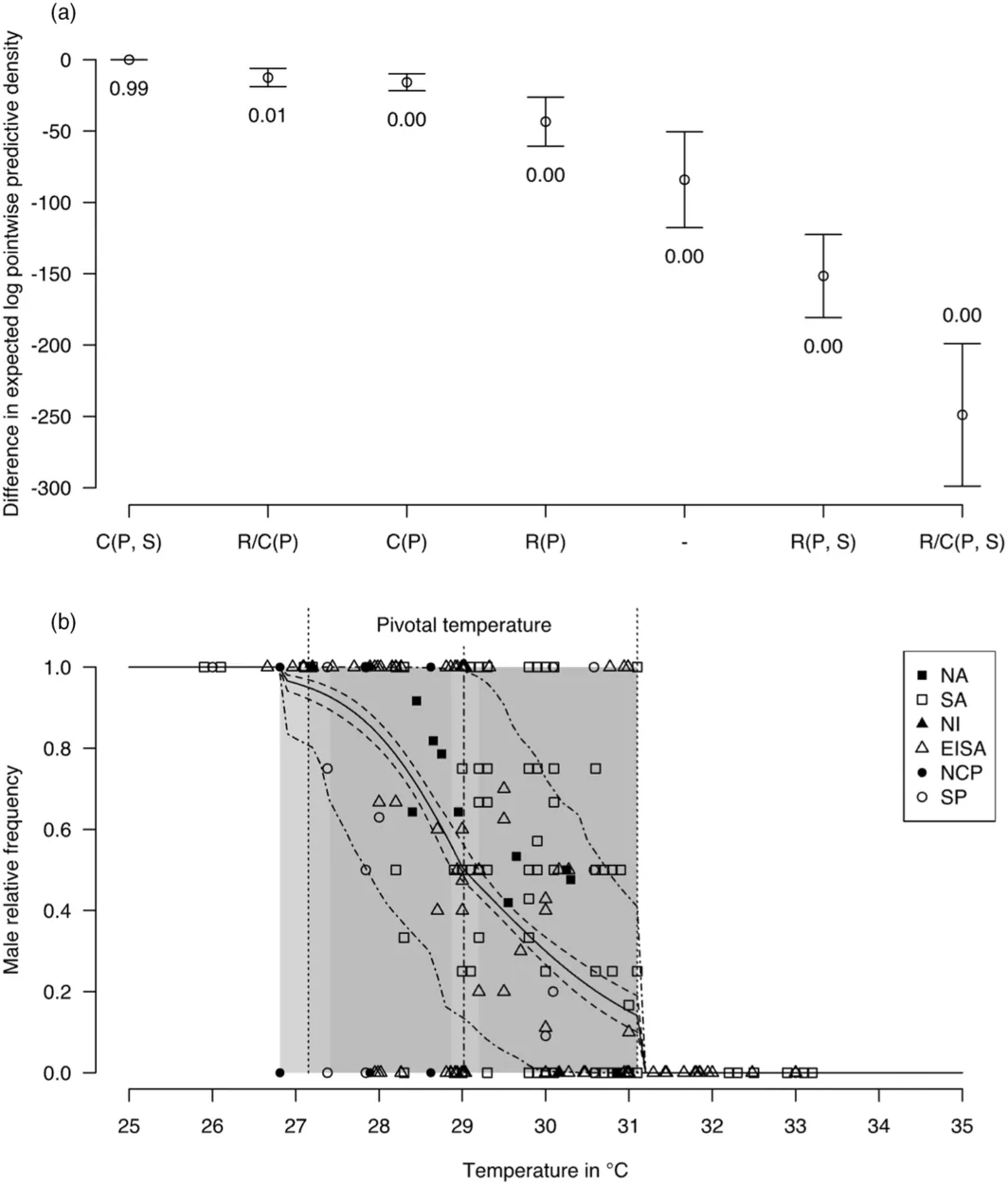
(a) Model selection using difference in expected log pointwise predictive density depending on random effects (R is for RMU, C is for Clutch and R/C is Clutch within RMU) included in a flexit** model. The parameters are in parenthesis (P or S [being SL + SH]) and represent how the random effects are applied. The bars are standard errors. The values are the probability that each model is the best among the tested models for predictive accuracy. (b) Bayesian fitted TSD pattern with flexit** model for 1480 eggs incubated at 107 constant temperatures. The plain curve is the median fixed effect model with the 95% credible envelop shown in dashed lines. The interrupted curves are the 95% credible model including the Clutch as random effect. The symbols represent the RMU origin of eggs (see text). The median estimate of the transitional range of temperatures (TRT) from the fixed‐effects component of the model is shown as a greyed rectangle with credible intervals of TRT and pivotal temperature limits shown as lighter greyed.

The fitted TSD thermal reaction norm is shown in Figure 2b. The selected model yielded a pivotal temperature (temperature producing 1:1 sex ratio) of 29.32°C (95% credible intervals 29.01°C; 29.67°C, Figure 2b). The fitted thermal reaction norm was asymmetrical, with slope parameters *SL* = 1.86°C (1.31°C; 2.75°C; range of temperatures from 0.05 to 0.5 sex ratio) and *SH* = 2.25°C (1.51°C; 3.31°C; range of temperatures from 0.5 to 0.95 sex ratio). The estimated transitional range of temperatures (TRT) was broad, at 4.15°C (3.56°C; 5.05°C). The limits of TRT are 27.46°C (26.78°C; 27.88°C) and 31.58°C (31.02°C; 32.45°C). All values are reported as medians with corresponding 95% credible intervals.

### 3.3 Model of embryo growth

The thermal reaction norm of embryo growth, based on the incubation duration of the 64 monitored nests, was fitted using the 4‐ and 6‐parameter Schoolfield et al. (1981) equations (Equation 2). The simplest 4‐parameter model was selected against the 6‐parameter model (probability being the nest model 0.84 vs. 0.16) using difference of expected log pointwise predictive density (Vehtari et al., 2017). The general pattern of the thermal reaction norm of embryo growth, based on the incubation of 64 nests, is similar to the ones observed for other marine turtles (Figure 3a). In general, nest temperatures were high. At low temperatures, there was a very slow rate of development. An optimum for developmental rate was reached at 31–32°C and above this temperature range, the developmental rate slows (embryo growth rates decrease at higher temperatures). An example of a modelled embryo straight carapace length (SCL) is shown in Figure 3b along with the recorded incubation temperatures. The TSP is located at the period when the embryo SCL (or equivalent SCL, relatively early in development when SCL is not yet measurable) is 13 mm to 41 mm and it spans 20 days.

**FIGURE 3:**
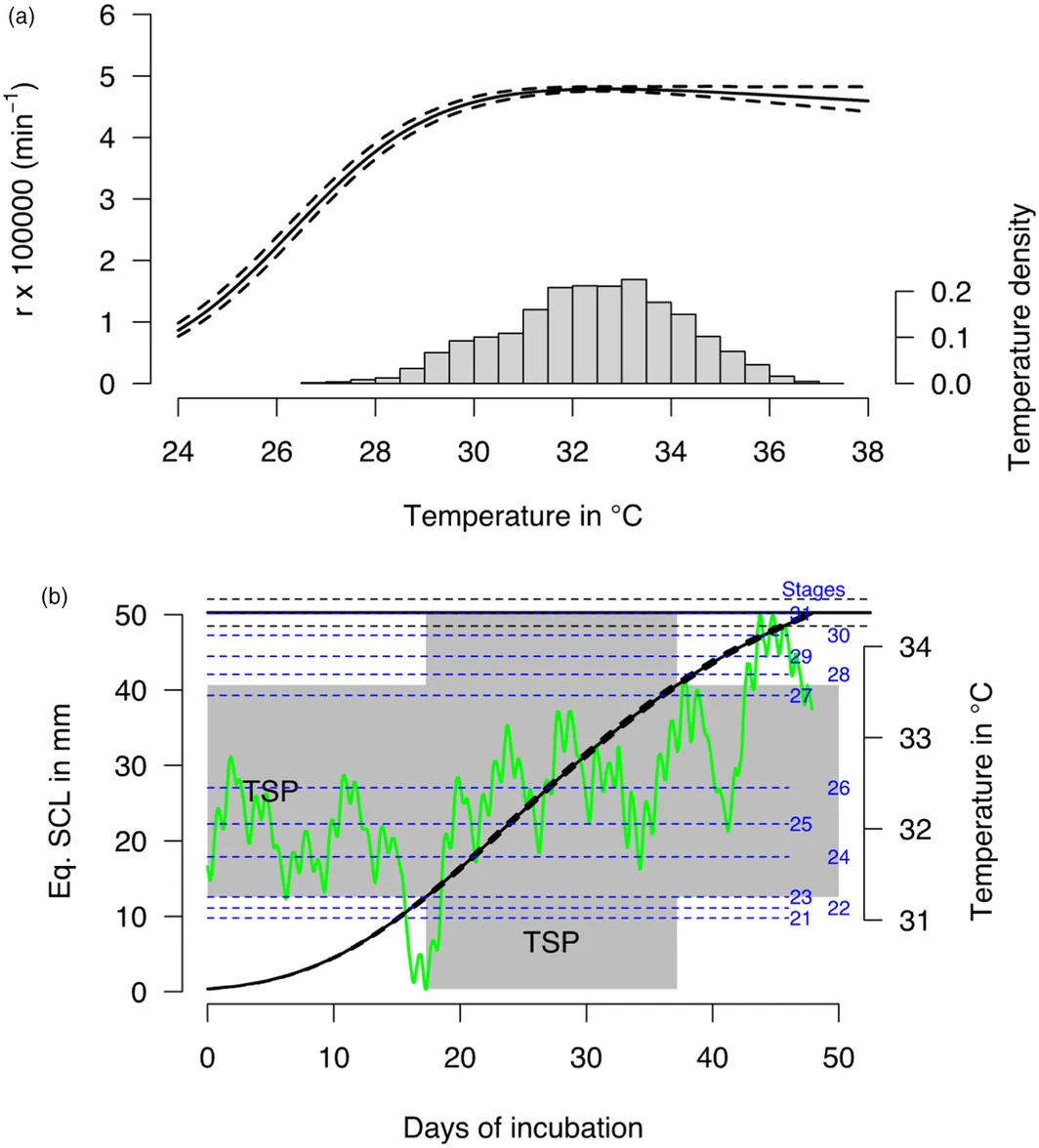
(a) The curves are the thermal reaction norm of the green turtle embryo growth modelled for the 64 nests monitored in Florida; r is the instantaneous growth rate min^−1^. The histograms identify the temperature bins. (b) Example of modelled embryo growth of equivalent SCL in mm, with the position of TSP in grey, based on embryological stages in blue (from stage 23 to middle of stage 27). The clutch thermal data is shown in green. In both (a and b), black solid curve is median and dashed lines are 95% posterior predictive credible interval.

### 3.4 Model for sex ratio

We fitted and tested nine different models for predicting sex ratios (Table 1). The selected model uses a growth‐weighted temperature average, a fitted thermal reaction norm of sexualization (*TRNS*) and sensitivity within the TSP (*S‐TSP*). The choice of a growth‐weighted average temperature is consistent with current experimental evidence on how TSD operates (Georges et al., 1994; Pieau, 1982). The probability that this model is the best among these eight models tested is 0.57. All the next three models in AICc ranking include the *TRNS* (Table 1, models 3, 6 and 7). Among the two effects of the selected model, the most important is the *TRNS* over the *S‐TSP*. However, adding *S‐TSP* to the *TRNS* model improved the prediction (∆AICc = 2.96).

**TABLE 1:** The nine tested models with the associated AICc and Akaike weight (probability that the model is the best among the tested models).

Model	Growth‐weight	*TRNS*	*S‐TSP*	*TSP*	AICc	∆AICc	Akaike weight
1	—				107.73	14.74	0.00
2	X				110.81	17.82	0.00
3	X	X			95.95	2.96	0.13
4	X		X		113.50	20.51	0.00
5	X			X	113.16	20.17	0.00
**6**	**X**	**X**	**X**		**92.99**	**0.00**	**0.57**
7	X	X		X	95.40	2.41	0.17
8	X		X	X	117.45	24.46	0.00
9	X	X	X	X	95.93	2.94	0.13

*Note*: The selected model is shown in bold. The column Growth‐weight indicates if the average temperature within the TSP used a growth‐weight scheme (X) or a time‐weight scheme (−). The *TRNS*, *S‐TSP* and *TSP* are effects considered with each model.

The thermal reaction norm of sexualization (TRNS) is very high at low temperatures (<29°C) indicating an enhanced effect of these low temperatures on sexualization of the gonad (Figure 4a). To illustrate this effect and clarify the concept of TRNS, consider a nest in which half the TSP occurs at 29°C (*TRNS* = 1.35; Figure 4a) and the other half at 31°C (*TRNS* = 0.17; Figure 4a). The constant‐temperature equivalent (CTE) within the TSP will be (29 × 1.35 + 31 × 0.17)/(1.35 + 0.17) = 29.22°C indicating that lower temperature (29°C) will have a stronger effect than higher temperature (31°C). Sensitivity to sexualization in the TSP (S‐TSP) increases constantly throughout the TSP until the end where we observe a sharp increase after Stage 26 (day 50 for this temperature profile) (Figure 4b). This may suggest that the end of the TSP is more important in sex determination than the start of this development window. Differences between the observed and predicted sex ratio are shown in Figure 4c. All points, except one, cross the 1:1 line taking into account the 95% confidence (for observations) or credible (for predictions) intervals. The proportion of observation‐prediction out of the 1:1 line (1/62 = 0.02) is very close, and even lower, to the expected value of 0.05.

**FIGURE 4:**
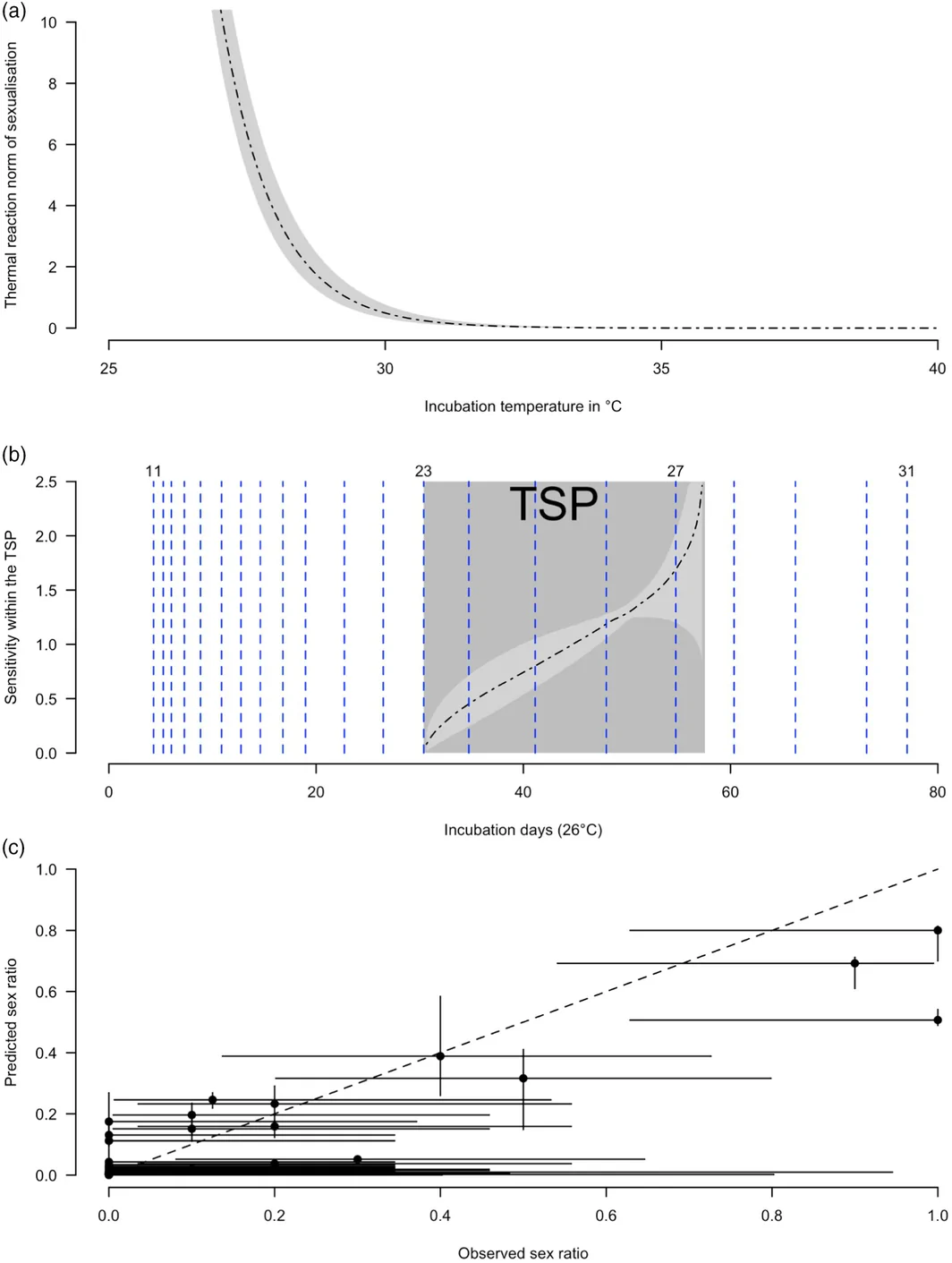
Fitted models of thermal reaction norm of sexualization (a) and sensitivity within the TSP (b). In both graphs, the 95% posterior predictive credible interval is shown in light grey and the median is shown as a dashed line. In B, the vertical dashed lines are the embryological stages based on Miller (1985) and the TSP is shown in dark grey. In (c) the observed sex ratios (male relative frequency) of the monitored nests are shown on the *x*‐axis and the posterior predictive predictions of the model are shown on the *y*‐axis. The *x*‐bars are the 95% confidence intervals of the observations, and the *y*‐bars are the 95% credible intervals of the predictions.

## 4 DISCUSSION

### 4.1 Temperature in green turtle nests in Florida

The nest temperatures recorded in this study are high, particularly when compared with the average air temperature measured at 2 m above‐ground from the ECMWF database (Figure 1a), or with nest temperatures reported in other studies of green turtles (Broderick et al., 2000; Calderón Peña & Azanza Ricardo, 2021; Cheng et al., 2015). To explain this difference, it is important to consider that nest temperature heat is mainly driven by solar irradiation (Girondot, 2026), which varies with latitude. Although air temperature is correlated with solar irradiation, it should not be considered a direct proxy for nest temperature (Girondot, 2026).

While the average air temperature at 2 m altitude is a biased indicator of the nest temperature, it shows that females nest during the hottest part of the season. This appears to be a general pattern in marine turtles (Mrosovsky & Provancha, 1992).

### 4.2 TSD thermal reaction norm

The selected model has a high pivotal temperature of 29.32°C (29.01°C; 29.67°C) and the upper limit of the TRT is 31.58°C (31.02°C; 32.45°C) which is near the upper range of observed field temperatures of green turtle nests (Broderick et al., 2000; Calderón Peña & Azanza Ricardo, 2021; Cheng et al., 2015).

The TRT was notably broad, at 4.15°C (3.56°C; 5.05°C) and within the TRT, the observed sex ratios are highly dispersed due to clutch effect and do not strictly follow the TSD pattern curve (Figure 2b). No RMU effect is detected. Together these lines of evidence indicate genetic and/or maternal differences among clutches in thermal sensitivity of sex ratio (Janzen et al., 2019) and suggest that this trait has the potential to respond to selection. This wide range in TRT may infer a high degree of thermal resilience for sex ratios in this species, allowing males to be produced at higher than expected temperatures. It is important to note that this assessment of the temperature‐dependent sex determination (TSD) pattern is based on constant incubation temperatures. Extrapolation of this pattern to natural, fluctuating temperature regimes must be approached with caution, as TSD responses are highly sensitive to the magnitude and dynamics of temperature variability (see below).

### 4.3 The thermal reaction norm of embryo growth

The thermal reaction norm of temperature shown in Figure 3a for green turtles is similar to the one fitted using only constant incubation temperatures for this species (Yao et al., 2022). It shows that the models of embryo growth at constant and fluctuating temperatures capture similar information.

The thermal reaction norm of embryo growth has been estimated for three other marine turtles previously. For leatherback sea turtles (*Dermochelys coriacea*), only temperatures from 25 to 32°C were available because at temperatures greater than 32°C mortality was very high (Rimblot et al., 1985). The growth rate is strictly increasing within this 25–32°C range (Girondot et al., 2024). For olive ridley sea turtles (*Lepidochelys olivacea*), the embryo growth rate was also increasing from 25°C up to 34°C but a decline was observed for temperatures higher than 34°C (Morales‐Mérida et al., 2018). Loggerhead sea turtles that nest at higher latitudes typically experience lower nest temperatures (Sénégas et al., 2009) than the other species or other loggerhead populations that nest in tropical and subtropical latitudes. Despite these cooler temperatures, the loggerheads exhibit an optimum embryo growth at 32°C and then a decline at warmer temperatures (Monsinjon et al., 2017). The green turtle embryo growth pattern shows an optimum around 31°C or 32°C and then declines slightly at warmer temperatures up to 38°C (Figure 3a). This result aligns with the observation of lower hatching success at constant 34°C temperature compared to 26, 28, 30 and 32°C observed by Yao et al. (2022), indicating that although embryos can survive at these temperatures, embryo suffers at temperatures >34°C. The main difference between Yao et al. (2022) study and our study is that in the former they maintained a constant temperature of 34°C, whereas the field nests are exposed to relatively short pulses of high temperature. These results suggest that embryos may be more resistant to intermittent short pulses of high temperature rather than prolonged exposure.

### 4.4 Sex ratio from time series of temperatures

The selected model with best sex ratio prediction without overparameterization includes thermal reaction norm of sexualization (*TRNS*) and sensitivity within the TSP (*S‐TSP*). The *TRNS* shows an unexpected pattern: it is very high for temperatures below 29°C and becomes low and nearly constant (but not zero) for temperatures above 30°C (Figure 4a). This means that (1) when lower (<29°C) and higher (>30°C) temperatures are within the TSP, the masculinizing temperatures will have the greatest influence on sexualization of the gonad. (2) It suggests that a repressor of feminization is synthesized at masculinizing temperatures and when both masculinizing and feminizing temperatures are present within the TSP, the repressor persists and masks the effect of high temperatures. From a mechanistic point of view, the oestrogens are the hormones involved in feminization of the gonad (Pieau & Dorizzi, 2004) and their absence masculinizes the gonad. The enzyme aromatase CPY19A1 functions to produce oestrogens from androgens but is not itself the temperature sensor (Milnes et al., 2002). It has already been proposed that aromatase expression is controlled by a repressor factor expressed at masculinizing temperatures rather than its enhancement at feminizing temperatures (Girondot et al., 2010). Recently, such a repressor has been identified. Temperature modulates an upstream regulator, the signal transducer and activator of transcription 3 (STAT3). STAT3 is a transcriptional regulator of histone demethylase *Kdm6b* (Weber et al., 2020) and KDM6B directly promotes the transcription of the male sex‐determining gene *dmrt1* (Ge et al., 2018).

The second most influencing effect to determine sex ratio is the sensitivity within the TSP (*S‐TSP*). Our results showed a non‐uniform and increasing importance of temperatures from the beginning of TSP to the end (Figure 4b). The limits of TSP as defined by experiments (from stage 23 to middle of stage 27) are confirmed here. The influence of temperature is smallest at the beginning of the TSP when the gonadal tissues begin to proliferate and differentiate. As embryonic growth proceeds through the TSP, the temperature influence increases and is maximum at the end of the TSP. This conclusion is logical based on the current knowledge of molecular functions for TSD, where an increasing number of genes are differentially expressed progressively throughout the TSP (Czerwinski et al., 2016).

The thermal reaction norm of sexualization, the sensitivity within the TSP and the thermal reaction norm of embryo growth work simultaneously to generate the thermal reaction norm of sex ratio. This set of interacting distinct processes explains the discordance that is sometimes observed between incubation duration and sex ratio (Fuentes et al., 2017).

### 4.5 The making of males

In our study, males are produced at supposedly feminizing temperatures. Furthermore, the male relative frequency per nest was 0.08 with a large standard deviation (0.22) that was never observed in 10,000 random replicates of a binomial distribution. For example, three nests have a male frequency >0.9 with 9 or 10 hatchlings sexed for each, which is highly unexpected based on random sampling with 0.08 male relative frequency. We identify four mechanisms that may generate males in a feminizing thermal world.

1. Our estimate of pivotal temperature is higher and transitional range of temperatures (TRT) is wider than previously estimated (Mrosovsky et al., 1984) because we have demonstrated the influence of a Clutch effect (female or male identity or other factors such as season or time of laying during season) rather than a RMU effect (Figure 2b).
2. The presence of a mechanism that amplifies the effect of lower temperatures (Figure 4a) suggests that when embryos are exposed to both low and high temperatures during the TSP, the influence of the lower temperatures is disproportionately increased. This may increase the masculinizing effect of cooler thermal conditions and interact with the effects of temperature on sex ratio and embryo growth rate.
3. The median heterogeneity of nest temperatures is 1.58°C (95% credible interval: 1.52–1.64°C) at the scale of the beach with a maximum heterogeneity of 2.16°C among nests (Figure 1b). This heterogeneity is in the same order of magnitude as the TRT; thus, the variation in nest temperatures is large enough to span the range that influences sex. As a result, depending on the nest, the temperature during the TRT could be masculinizing or feminizing because of this variation.
4. The temporal autocorrelation of nest temperatures is large, with a lag of 17 days (Figure 1c). For example, if the temperature is low at one time point during incubation, effectively it will stay low for an average of 17 days. This is approximately the duration of the TSP, so depending on timing, this decrease in temperature could potentially masculinize the sex ratio. Without this autocorrelation, the average temperature remaining high means that only feminized nests will be produced, yet the empirical data do not support such a result.

## 5 CONCLUSIONS

Our results indicate that green turtles have some capacity to produce males even under predominantly feminizing thermal conditions. This capacity for male production appears to arise from a combination of factors, including environmental heterogeneity across nests, strong temporal autocorrelation in nest temperatures and non‐linear thermal sensitivity during the thermosensitive period. The unexpectedly wide TRT and an elevated pivotal temperature suggest that previous estimates may have underestimated the species' potential for male production under natural conditions. Moreover, our model reveals that masculinizing temperatures exert a disproportionately strong influence when present, particularly during late stages of the TSP, pointing to complex underlying mechanisms of sex differentiation beyond mean nest temperature alone.

By integrating mechanistic models of both embryo growth and sexual differentiation, this study offers the first validated framework for estimating sex ratios in *Chelonia mydas* directly from field‐recorded thermal profiles. These insights not only improve our understanding of the demographic consequences of climatic shifts in species that display TSD but also provide a valuable predictive tool to inform targeted conservation actions, such as timing of nest relocations, artificial shading or habitat prioritization. As the frequency and intensity of climate extremes increase, such predictive capacity becomes essential to anticipate shifts in reproductive output and support the long‐term viability of sea turtle populations worldwide.

## AUTHOR CONTRIBUTIONS

Marc Girondot: Conceptualization, data curation, formal analysis, methodology, software, validation, visualization, writing—original draft preparation, and writing—review and editing. Emily Turla: Conceptualization, data curation, investigation, methodology, validation, and writing—review and editing. Kirsty Scott: Formal analysis, validation, writing—original draft preparation, and writing—review and editing. Jeanette Wyneken: Conceptualization, data curation, formal analysis, funding acquisition, investigation, methodology, project administration, resources, supervision, validation, writing—original draft preparation, and writing—review & editing.

## CONFLICT OF INTEREST STATEMENT

The authors declare that they have no conflicts of interest.

## Supporting information


**Supporting Information S1.** List of monitored nests.

**Supporting Information S2.** The intersexes.
**Supporting Information S3.** Data of sexed green turtles incubated at constant incubation temperatures.
**Supporting Information S4.** Common statistical procedures and list of parameters.

## Data Availability

All workflows with detailed script parameters as well as datasets are available on GitHub at https://github.com/Marc‐Girondot/TSD_Cm.
