# Supplementary material for: Male production in green sea turtles under a feminizing climate: A validated model of sex ratios with temperature‐dependent sex determination
Source: J Anim Ecol. 2026 Aug 11;95(10):2119–32. doi: 10.1111/1365-2656.70334 (PMC13643197; doi:10.1111/1365-2656.70334)
Supplement: Supplementary file 1 [file JANE-95-2119-s001.docx]

**Supplementary material 1: List of monitored nests.**

**See csv file.**

**Supplementary material 2: The intersexes**

A total of 24 intersexes were described in four studies for eggs incubated at 11 constant temperatures (range 27°C to 33°C). An intersex individual may refer to individuals with mismatching gonadal structure and sexual ducts. Or for example, an individual with the presence of both medullary cords (characteristic of testes) and a developed cortex with numerous germ cells at meiotic prophase (characteristic of ovaries) within the same gonad, known as an ovotestis. Only the former case should be considered here as the temperature acts on the gonad itself (Pieau & Dorizzi, 2004) and the differentiation of the ducts is a consequence of gonadal differentiation.

Miller and Limpus (1981) described nine intersexes out of 65 embryos incubated at 26°C, 29°C and 33°C. The presence of intersexes for eggs incubated at 26°C and 33°C is quite unexpected as these are 100% masculinizing and feminizing temperatures respectively for *Chelonia mydas*. However, it is not possible to conclude, based on the text, if these intersexes have ovotestes or mismatch between ducts and gonads. All these intersexes have been removed for the analysis.

In ROSIE database v. 1.0.3, seven intersexes are wrongly indicated from the Syed and Mazlan (2004) study. Indeed, based on the original publication, they were not intersex, but these individuals had indeterminate gonads: “Whereas at the temperature 27.2 ºC and 27.7ºC, 20% and 16% respectively are indeterminate due to poor technique on preparation of specimen slides.” Data have been corrected here.

Other green turtle intersexes come from two publications from the same laboratory (Godfrey & Mrosovsky, 2006, Mrosovsky *et al.*, 1984). Based on the descriptions, these gonads were typical ovotestes: “For intersex, the germinal epithelium had to extend more than halfway around the gonad and be separated from the medulla by a clear tunica albuginea.” (Mrosovsky *et al.*, 1984). *Emys orbicularis* (freshwater turtle) gonads from eggs incubated at pivotal temperature were studied at hatching and at 1, 4, and 9 months after hatching (Girondot *et al.*, 1998). At hatching, oocytes in ovotestes begin to degenerate into the cortex. Degeneration continues after hatching, so ovotestes generally evolve into typical testes (Pieau & Dorizzi, 2004). Based on these observations, these individuals have been counted here as males.

**Bibliography**

Girondot M, Fouillet H, Pieau C (1998) Feminizing turtle embryos as a conservation tool. *Conservation Biology,* **12**, 353-362.

Godfrey MH, Mrosovsky N (2006) Pivotal temperature for green sea turtles, *Chelonia mydas*, nesting in Suriname. *Herpetological Journal,* **16**, 55-61.

Miller JD, Limpus CJ (1981) Incubation period and sexual differentiation in the green turtle *Chelonia mydas* L. In: *Proceedings of the Melbourne Herpetological Symposium.* (eds Banks CB, Martin AA) pp 66-73, The Royal Melbourne Zoological Gardens, Zoological Board of Victoria, Parkville.

Mrosovsky N, Dutton PH, Whitmore CP (1984) Sex ratios of two species of sea turtle nesting in Suriname. *Canadian Journal of Zoology,* **62**, 2227-2239.

Pieau C, Dorizzi M (2004) Oestrogens and temperature-dependent sex determination in reptiles: all is in the gonads. *Journal of Endocrinology,* **181**, 367-377.

Syed A, Mazlan I (2004) Temperature dependent sex determination and hatching performance of green turtle (*Chelonia mydas*) at Chendor Rookery on the east coast of Peninsular Malaysia. In: *Proceedings of the International Symposium on SEASTAR2000 and Bio-logging Science (The 5th SEASTAR2000 Workshop).* pp 11-15.

**Supplementary materials 3: Data of sexed green turtles incubated at constant incubation temperatures.**

| Total.Sexed | Total.Temperatures | Total.Clutches | RMU.2023 | Source |
| --- | --- | --- | --- | --- |
| 251 | 12 | 3 | East Indian and Southeast Asia | 1 |
| 161 | 37 | 9 | East Indian and Southeast Asia | 2 |
| 61 | 4 | 1 | East Indian and Southeast Asia | 3 |
| 108 | 4 | 1 | North Atlantic | 4 |
| 81 | 7 | 1 | North Atlantic | 5 |
| 55 | 2 | 1 | Northwest Indian | 6 |
| 391 | 27 | 33 | South Atlantic | 7 |
| 99 | 4 | 4 | Southwest Pacific | 8 |
| 94 | 3 | 1 | Southwest Pacific | 9 |
| 49 | 2 | 1 | Southwest Pacific | 10 |
| 49 | 2 | 1 | Southwest Pacific | 11 |
| 81 | 3 | 12 | North Central Pacific | 12 |

1: Syed, A., & Mazlan, I. (2004). Temperature dependent sex determination and hatching performance of green turtles (*Chelonia mydas*) at Chendor Rookery on the east coast of Peninsular Malaysia. Proceedings of the International Symposium on SEASTAR2000 and Bio-logging Science, 2004, 11-15.

2: Bentley, B. P., Stubbs, J. L., Whiting, S. D., & Mitchell, N. J. (2020). Variation in thermal traits describing sex determination and development in Western Australian sea turtle populations. Function Ecology, 34(11), 2302-2314.

3: Tokunaga, S. (1991). A preliminary observation on temperature-dependent sex determination in two species of Japanese sea turtles. Umigawe Newsletter of Japan, 7, 3-7.

4: Mrosovsky, N., Dutton, P. H., & Whitmore, C. P. (1984). Sex ratios of two species of sea turtle nesting in Suriname. Canadian Journal of Zoology, 62(11), 2227-2239.

5: Godfrey, M. H., & Mrosovsky, N. (2006). Pivotal temperature for green sea turtles, *Chelonia mydas*, nesting in Suriname. Herpetological Journal, 16(1), 55-61.

6: AlKindi, A. Y. A. (2005). Circulating hormone levels in hatchlings of green turtle, Chelonia mydas, from Ras Al-Hadd Reserve, Oman. Sultan Qaboos University Journal For Science, 10(1), 43-50. doi: 10.24200/squjs.vol10iss0pp43-50

7: Tilley, D., Ball, S., Ellick, J., Godley, B. J., Weber, N., Weber, S. B., & Broderick, A. C. (2019). No evidence of fine scale thermal adaptation in green turtles. Journal of Experimental Marine Biology and Ecology, 514-515, 110-117.

8: Podreka, S., Georges, A., Maher, B., & Limpus, C. J. (1998). The environmental contaminant DDE fails to influence the outcome of sexual differentiation in the marine turtle *Chelonia mydas*. Environmental Health Perspectives, 106(4), 185-188.

9: Burgess, E. A., Booth, D. T., & Lanyon, J. M. (2006). Swimming performance of hatchling green turtles is affected by incubation temperature. Coral Reefs, 25(3), 341-349.

10: Porter, E., Booth, D. T., Limpus, C. J., Staines, M. N., & Smith, C. E. (2021). Influence of short-term temperature drops on sex-determination in sea turtles. Journal of Experimental Zoology Part A, 335(8), 649-658.

11: Miller, J. D., & Limpus, C. J. (1981). Incubation period and sexual differentiation in the green turtle *Chelonia mydas* L. In C. B. Banks & A. A. Martin (Eds.), Proceedings of the Melbourne Herpetological Symposium (pp. 66-73). The Zoological Board of Victoria, Parkville.

12: Nelson, T., Estes, J., & Wibbels, T. (2007). Histological evaluation of hatchling sex ratios of Hawaiian green sea turtles. Inquiro, 2007(1), 27-31.

**Supplementary material 4: Common statistical procedures and list of parameters**

Models fitting

The models were fitted using the Metropolis-Hastings algorithm, employing a Markov chain generated through a Monte Carlo sampling method (Hastings, 1970, Metropolis *et al.*, 1953) using Bayesian scheme. Chain stability and convergence were assessed using the diagnostics proposed by Gelman and Rubin (1992) and Geweke (1992), respectively.

Model Validation

Leave-One-Out cross-validation (LOO) was used to estimate the out-of-sample predictive accuracy of the Bayesian models (Vehtari *et al.*, 2017). This approach evaluates how well the model predicts new data, under the assumption that the new data come from the same distribution as the observed data. Pareto smoothed importance sampling (PSIS-LOO) was used to approximate LOO efficiently, to avoid the need to refit the model repeatedly. The Pareto-𝑘̂ statistic serves as a diagnostic for the reliability of the PSIS-LOO estimates. The Expected Log Predictive Density (ELPD) is the primary metric used in LOO, quantifying how well the model predicts unseen data and supporting model selection based on predictive performance.

The ELPD provides an information-theoretic criterion that balances goodness of fit with model complexity, effectively penalizing models that overfit the data (Vehtari, Gelman & Gabry 2017). To account for uncertainty, simulation using the pointwise LOO log-predictive densities was used to preserve the correlation between models, because all models are evaluated on the same observations.

This method can be considered a Bayesian analogue of the Akaike Information Criterion (AIC) (Akaike, 1974), as it integrates over the posterior distribution to estimate predictive performance.

List of parameters

*flexit*** model for TSD pattern; equation 1: *P*, *SL*, *SH*, *TRT*, *l*

Thermal reaction norm of embryo growth; equation 2: $T$, ${\Delta H}_{A}^{\neq}$, ${\Delta H}_{H}$, $T_{{1/2}_{H}}$, $\rho_{(298 K)}$

Gompertz growth model; equation 3: *X*(*t*), *T*, *t*, *X*(0), *r*(*T*), *K*

Thermal reaction norm of sexualisation (*TRNS*); equation 2: $T$, ${TRNS.\Delta H}_{A}^{\neq}$, ${TRNS.\Delta H}_{H}$, $TRNS.T_{{1/2}_{H}}$; ${TRNS.\rho}_{(298 K)}$ is fixed to 100

Sensitivity within the TSP (*S-TSP*): *shape1*, *shape2*

Limits of the TSP: *BeginTSP*, *EndTSP*

Constant temperature equivalent; equation 4: CTE

**Bibliography**

Akaike H (1974) A new look at the statistical model identification. *IEEE Transactions on Automatic Control,* **19**, 716-723.

Gelman A, Rubin DB (1992) Inference from iterative simulation using multiple sequences. *Statistical Science,* **7**, 457-511.

Geweke J (1992) Evaluating the accuracy of sampling-based approaches to calculating posterior moments. In: *Bayesian Statistics.* (eds Bernado JM, Berger JO, Dawid AP, Smith AFM). Oxford, UK, Clarendon Press.

Hastings WK (1970) Monte Carlo sampling methods using Markov chains and their applications. *Biometrika,* **57**, 97-109.

Metropolis N, Rosenbluth AW, Rosenbluth MN, Teller AH, Teller E (1953) Equations of state calculations by fast computing machines. *Journal of Chemical Physics,* **21**, 1087-1092.

Vehtari A, Gelman A, Gabry J (2017) Practical Bayesian model evaluation using leave-one-out cross-validation and WAIC. *Statistics and Computing,* **27**, 1413-1432.
